# Cloning, 3D Modeling and Expression Analysis of Three Vacuolar Invertase Genes from Cassava (*Manihot Esculenta* Crantz)

**DOI:** 10.3390/molecules19056228

**Published:** 2014-05-15

**Authors:** Yuan Yao, Xiao-Hui Wu, Meng-Ting Geng, Rui-Mei Li, Jiao Liu, Xin-Wen Hu, Jian-Chun Guo

**Affiliations:** 1 Key Laboratory of Biology and Genetic Resources of Tropical Crops, Ministry of Agriculture, Institute of Tropical Bioscience and Biotechnology, Chinese Academy of Tropical Agricultural Sciences, Haikou 571101, China; E-Mails: yaofaraway1@163.com (Y.Y.); ficz@163.com (X.-H.W.); liruimei@itbb.org.cn (R.-M.L.); liujiao@itbb.org.cn (J.L.); 2 Agricultural College, Hainan University, Haikou 571104, China; E-Mail: mengtinggeng8908@163.com

**Keywords:** cassava, expression analysis, molecular cloning, vacuolar invertase, 3D modeling

## Abstract

Vacuolar invertase is one of the key enzymes in sucrose metabolism that irreversibly catalyzes the hydrolysis of sucrose to glucose and fructose in plants. In this research, three vacuolar invertase genes, named *MeVINV1-3*, and with 653, 660 and 639 amino acids, respectively, were cloned from cassava. The motifs of NDPNG (β-fructosidase motif), RDP and WECVD, which are conserved and essential for catalytic activity in the vacuolar invertase family, were found in *MeVINV1* and *MeVINV2*. Meanwhile, in *MeVINV3*, instead of NDPNG we found the motif NGPDG, in which the three amino acids GPD are different from those in other vacuolar invertases (DPN) that might result in MeVINV3 being an inactivated protein. The N-terminal leader sequence of MeVINVs contains a signal anchor, which is associated with the sorting of vacuolar invertase to vacuole. The overall predicted 3D structure of the MeVINVs consists of a five bladed β-propeller module at N-terminus domain, and forms a β-sandwich module at the C-terminus domain. The active site of the protein is situated in the β-propeller module. MeVINVs are classified in two subfamilies, α and β groups, in which α group members of *MeVINV1* and *2* are highly expressed in reproductive organs and tuber roots (considered as sink organs), while β group members of *MeVINV3* are highly expressed in leaves (source organs). All *MeVINVs* are highly expressed in leaves, while only *MeVINV1* and *2* are highly expressed in tubers at cassava tuber maturity stage. Thus, *MeVINV1* and *2* play an important role in sucrose unloading and starch accumulation, as well in buffering the pools of sucrose, hexoses and sugar phosphates in leaves, specifically at later stages of plant development.

## 1. Introduction

In most plant species, the product of photosynthesis is transported from source to sink organs in the form of sucrose [1]. The utilization of sucrose for various carbohydrate metabolic pathways depends on its cleavage into hexoses, and in higher plants either sucrose synthase (EC 2.4.1.13) or invertase (EC 3.2.1.26) catalyze this process [2]. Sucrose synthase reversibly catalyzes the conversion of sucrose into nucleoside diphosphate glucose and fructose [3]. Invertase irreversibly hydrolyzes sucrose into glucose and fructose [4]. Sucrose and its decomposition products play a key role in plant growth and development, carbohydrate storage, sugar signal transduction, biotic and abiotic stress responses, and gene regulation [5,6].

In higher plants, invertase presents a group of isozymes with different biochemical properties [7]. They are divided into two families, which are characterized as acidic or alkaline/neutral invertases by subcellular localization (cell wall, vacuole, cytosol, plastid or mitochondria), solubility (soluble or insoluble), optimum pH (acid or neutral/alkaline) and isoelectric point (pI) [8,9]. Cell wall invertases (insoluble) and vacuolar invertases (soluble) are labeled as acidic invertases due to their acidic optimum pH, encoded by a gene family originating from respiratory eukaryotes and aerobic bacteria [10]. Alkaline/neutral invertase (located in cytosol, plastid or mitochondria) is a group of soluble invertases with an alkaline/neutral optimum pH, originating from cyanobacteria [9,11]. Cell wall invertase is thought to function in regulation of sucrose partitioning [12], in response to wounding and pathogen infection [13], and in the regulation of seed and pollen development [14,15], while the proposed functions of the vacuolar invertases include the control of sugar composition in fruits and storage organs [16], osmoregulation and cell enlargement [17], response to drought stress [18], hypoxia and gravitropism [19]. In recent years, researchers have found that alkaline/neutral invertases also function in sugar signal transduction [20], the carbon balance between cytoplasm and organelles [9], and the starch synthesis of plastid [21]. The functions of plant invertase are diverse, thus, the dissection of the functions of invertase isoforms may help to clearly understand sucrose metabolism in plants.

Cassava is an important food source in the tropical and subtropical regions and feeds at least 500 million people in Africa, Latin America and Asia [22,23]. Its tuberous roots are rich in starch. Cassava is classified as a C3 plant, but its photosynthetic characteristics are similar to the C4 plants. The source organ (leaves) of cassava synthesizes large amounts of carbohydrates; however, its sink organ (tuberous roots) accumulates a small amount of carbohydrates, much lower than the theoretical value. Ihemere *et al.* reported that the targeted modification of enzymes regulating the source–sink relationship can increase both tuberous root number and total tuberous root biomass [24]. The vacuolar invertase in plants plays a key role in the source–sink relationship [25]. For instance, carrot plants expressing antisense mRNA of vacuolar invertase (GenBank accession number: X75351) had more leaves than the control plants, but the tap roots were smaller, and the ratio of leaf-to-root was higher; the carbohydrate content was elevated in leaves, while reduced in roots [12]. In leaves, the futile cycling of sucrose has a buffering effect on the pools of sucrose, hexoses and sugar phosphates, in which the vacuolar invertase is involved in this process [26,27]. Nagele *et al.* reported that the reduction of vacuolar invertase activity in plants caused a decline in photosynthesis and a reduced export of carbon to the associated metabolic pathways and sink organs (e.g., roots) [25]. In cassava, the genes of vacuolar invertase and their functions are not reported. In the present study, three vacuolar invertase genes from cassava were cloned according to the released sequence of the cassava genome. We investigated the evolutionary relationships, exon-intron structure, motif distribution and protein 3D structure of all family genes. To elucidate the possible roles of the vacuolar invertase genes, the spatial and tissue differential expressions of these genes were investigated during plant and tuber root development in source and sink organs. These results will be helpful to further understand the possible roles of vacuolar invertase in sucrose metabolism in cassava.

## 2. Results and Discussion

### 2.1. Cloning and Sequence Analysis of MeVINVs

**Figure 1:**
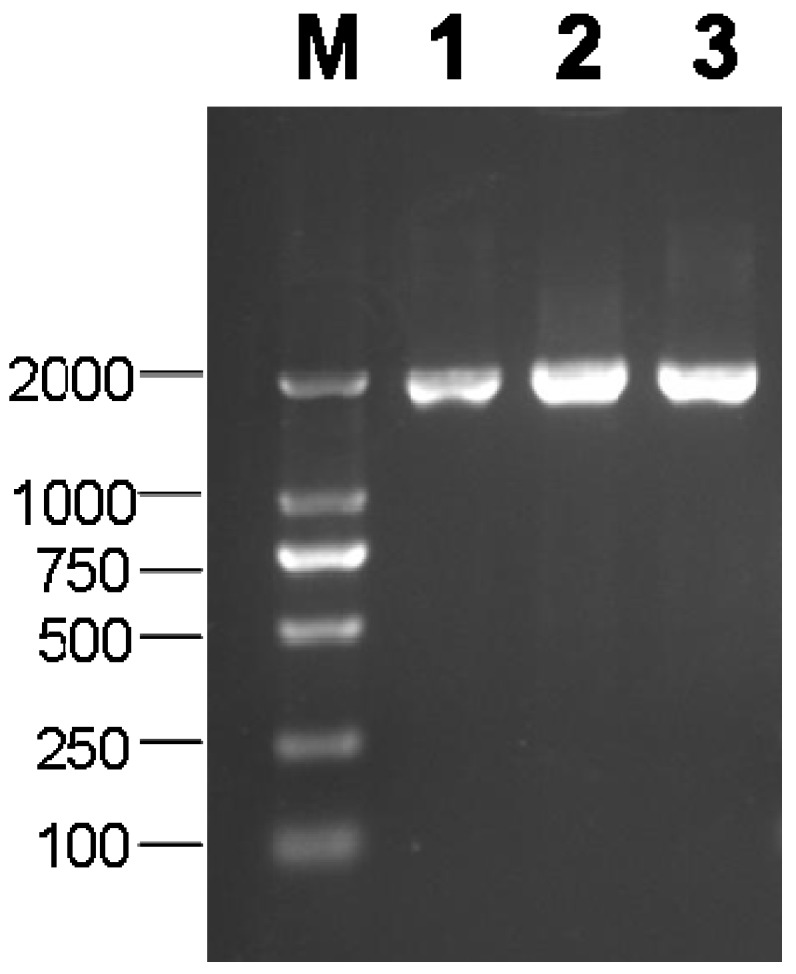
PCR product of *MeVINVs* (M: 2000 bp DNA marker). Lanes 1–3: PCR product of *MeVINV1-3*, respectively.

BLAST analysis of genome database of cassava identified three putative vacuolar invertase genes in the cassava genome. Based on the predicted sequence in the cassava genome, the gene-specific primers were designed and used to amplify the potential *MeVINVs* cDNA sequence from the leaves of the cassava by RT–PCR (Figure 1).

The cDNA and the deduced amino acid sequences of the *MeVINVs* described in this study were deposited in GenBank under the following accession numbers: *MeVINV1* (JX291158), *MeVINV2* (JQ792174), *MeVINV3* (JQ792173) (Table 1). The ORF length of the three genes are between 1920 and 1983 bp; their deduced amino acids are 653, 660, 639; and their theoretical pIs are 5.75, 5.26 and 4.60 for MeVINV1, MeVINV2 and MeVINV3, respectively (Table 1).

**Table 1:** Basic information of three cassava vacuolar invertase genes (*MeVINVs*).

Gene Name	Accession Number	Genomic Location	ORF Length (bp)	Length (aa)	pI	MW(kDa)
*MeVINV1*	JX291158	scaffold07035: 858738–862530	1962	653	5.75	73.3
*MeVINV2*	JQ792174	scaffold11581: 118337–121973	1983	660	5.26	73.5
*MeVINV3*	JQ792173	scaffold01127: 398782–401926	1920	639	4.60	71.5

**Figure 2:**
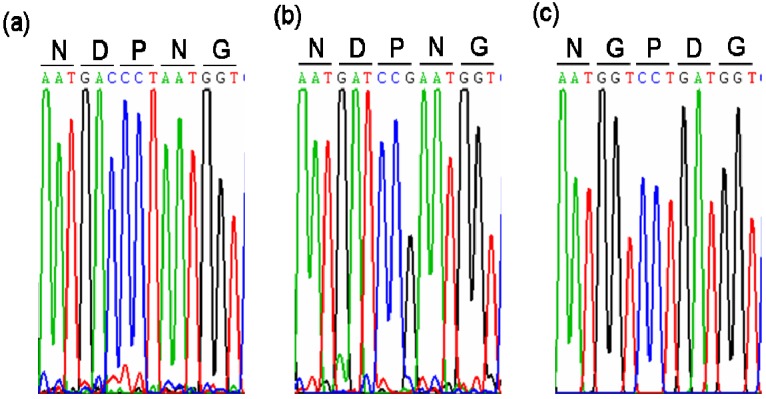
The chromatograms from the sequence data of the beta-fructosidase motif region of cassava vacuolar invertase. (**a**) β-Fructosidase motif region of MeVINV1; (**b**) β-Fructosidase motif region of MeVINV2; (**c**) β-Fructosidase motif region of MeVINV3.

**Figure 3:**
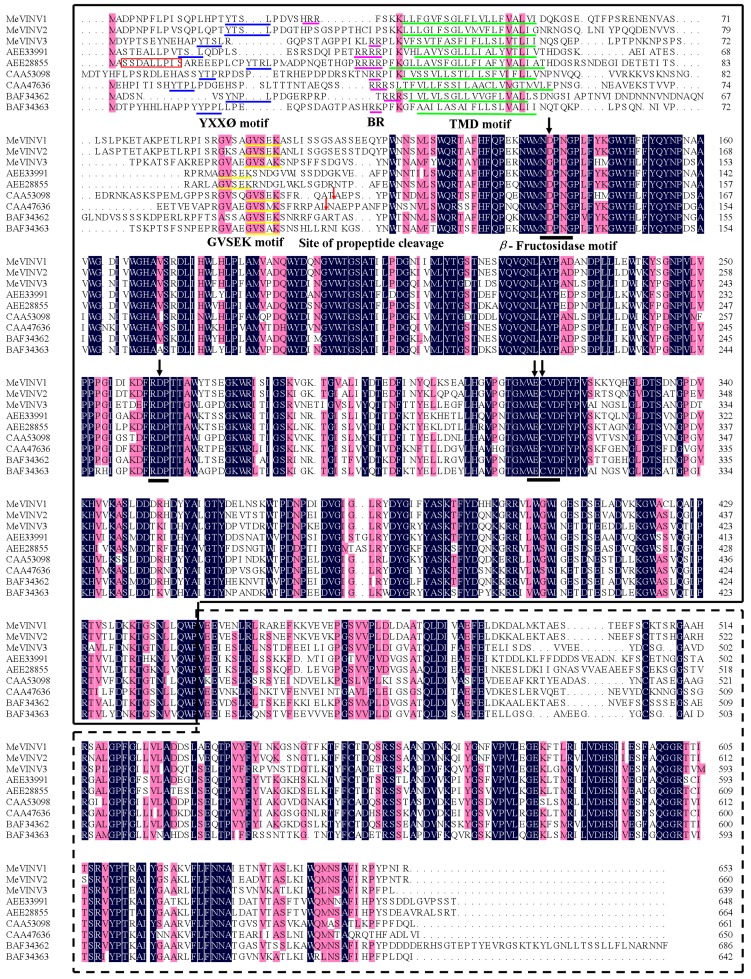
Alignment of the cassava vacuolar invertase amino acid sequences with amino acid sequences from three species. Identical amino acid residues in this alignment are shaded in black, and 75% or more similar amino acid residues are shaded in red. Black lines indicate the motifs which include active sites. Four arrows show the amino acids, which correspond with the active residues of invertase. The blue lines indicate the possible Basic region (BR) motifs. The green lines indicate the predicted transmembrane domain (TMD). Red box indicates the dileucine consensus motif for AtVI2 (AEE28855) [28]. The site of propeptide cleavage is indicated by red arrows. The N-terminus domain sequence is shown in the box (solid line), and the C-terminus domain sequence is also shown in the boxed (dashed line).

The deduced protein sequence of MeVINV1 shares 84.72%, 57.49% identity with MeVINV2,MeVINV3, respectively, while there is 58.89% identity share between MeVINV2 and MeVINV3. Multiple comparison of the deduced amino acid sequences of MeVINVs with the reported vacuolar invertase in *Arabidopsis thaliana* (AEE33991, AEE28855), *Daucus carota* (CAA53098, CAA47636) and *Citrus sinensis* (BAF34362, BAF34363) using the DNAMAN 6.0 program showed that the motifs NDPNG (β-fructosidase motif), RDP and WECVD that are conserved and essential for catalytic activity in the vacuolar invertase family were found in *MeVINV1* and *2*, but in *MeVINV3* the motifs were NGPDG, RDP and WECPD (Figure 2 and Figure 3). Analysis with the TMHMM Server v. 2.0 predicted that the invertase forms a signal anchor at N-terminal sequence. The predicted transmembrane domain (TMD) consists of amino acids 18–23. The basic region (BR) motif was identified in the N-terminal region of MeVINV1 and 2 (Figure 3). These motifs are associated with the sorting of vacuolar invertase to vacuole [28]. The N-terminal amino acid sequence of the mature vacuolar invertase polypeptide from *D. carota* (CAA53098, CAA47636) has been experimentally determined. The site of propeptide cleavage is generally locates upstream of β-fructosidase motif (22–31 amino acids) [29]. The likely site for proteolytic cleavage in cassava vacuolar invertase is unclear.

### 2.2. Structure Analysis of the MeVINV Family Genes

**Figure 4:**
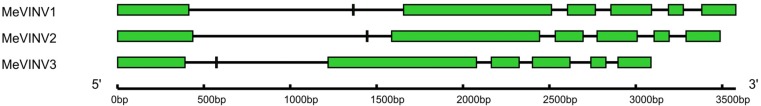
Exon-intron structures of three cassava vacuolar invertases in the cassava genome. Introns are shown as black lines, exons are shown as green boxes.

Alignment analysis of the full length cDNA sequences with the genomic sequences from the cassava genome database showed that all three vacuolar invertase family genes consist of seven exons in their coding sequences, and three amino acids (DPN or GPD) are located in the second exon, which is the smallest exon known in plants (Figure 4). The first intron in *MeVINV1* and *MeVINV 2* is the longest; however, the second intron is longest in *MeVINV3*.

### 2.3. Phylogenetic Analysis of MeVINVs

**Figure 5:**
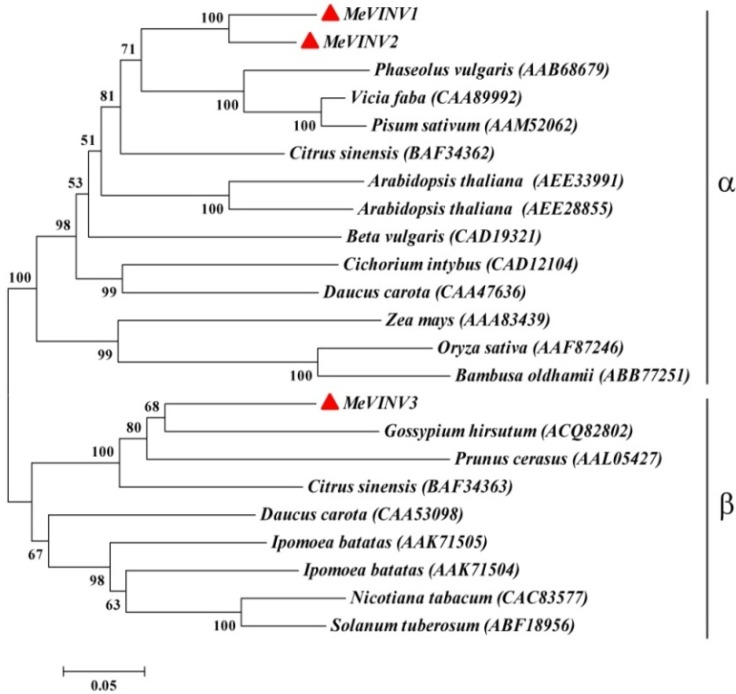
Phylogenetic relationship of the cassava vacuolar invertase amino acid sequences with amino acid sequences from fifteen species. The analysis was performed using the MEGA 5.2 program. The phylogenetic tree was constructed using the neighbor-joining method and bootstrapped 1000 times. The scale bar indicates the evolutionary distance between the groups. MeVINVs are shown in red triangles.

We compared the phylogenetic relationship of the MeVINVs in cassava with the vacuolar invertase genes from other plants based on their amino acid sequences using the MEGA5.2 program. The plant species and protein accession numbers used for the alignment and phylogenetic tree construction were as follows: *A. thaliana* (AEE33991, AEE28855), *D. carota* (CAA53098, CAA47636) and *C. sinensis* (BAF34362, BAF34363), *Ipomoea batatas* (AAK71504, AAK71505), *Phaseolus vulgaris* (AAB68679), *Cichorium intybus* (CAD12104), *Vicia faba* (CAA89992), *Pisum sativum* (AAM52062), *Beta vulgaris* (CAD19321), *Zea mays* (AAA83439), *Oryza sativa* (AAF87246), *Bambusa oldhamii* (ABB77251), *Gossypium hirsutum* (ACQ82802), *Prunus cerasus* (AAL05427), *Nicotiana tabacum* (CAC83577), *Solanum tuberosum* (ABF18956). The result showed that all vacuolar invertases in plants are classed in two main groups (α and β groups) (Figure 3). The vacuolar invertases in cassava, MeVINV1 and MeVINV2, belong in the α group and have a close relationship with the vacuolar invertases of AAB68679 in *P. vulgaris*, CAA89992 in *V. faba* and AAM52062 in *P. sativum* (AAM52062). Meanwhile the *MeVINV3* belongs in the β group and has a close relationship with the vacuolar invertases of ACQ82802 in *G. hirsutum*, AAL05427 in *P. cerasus*, and BAF34363 in *C. sinensis* (Figure 5).

### 2.4. Three-Dimensional (3D) Structure of MeVINVs

**Figure 6:**
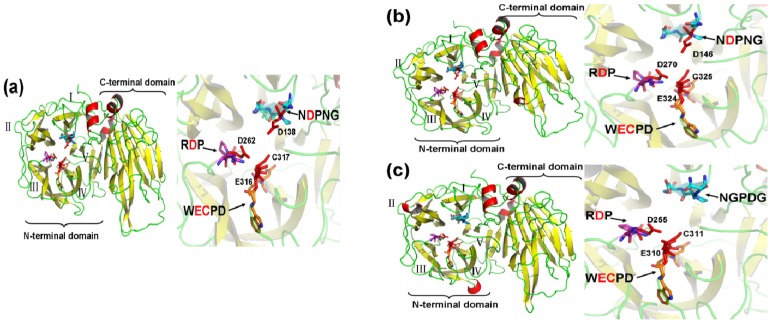
The cartoon representation and active site of the predicted 3D structure model of MeVINVs. Roman numerals (I–V) show the five blades of the β-propeller module, respectively. The graphics at the right side show a close-up view of the active site. The motifs (NDPNG or NGPDG, RDP, and WECPD) are shown as sticks, and the red sticks indicate the catalytic residues. Single-letter amino acid abbreviations are used with position numbers.

The three-dimensional (3D) structure of MeVINVs were modeled based on the X-ray structure of *Pachysandra terminalis* 6-fructosyltransferase protein (Protein Databank ID 3ugg; vacuolar invertase was its ancestor) using SwissModel [30]. The 6-fructosyltransferase showing 66.67%, 67.16%, and 64.87% sequence identity for *MeVINV1*, *MeVINV2*, and *MeVINV3*, respectively, and was thus determined to be the best template for model construction. The overall predicted structure of MeVINVs reveals a similar model with plant acid invertase: their N-terminal domain consists of a β-propeller module, while the C-terminal domain is formed by two β-sheets, called a β-sandwich module (Figure 6). The β-propeller module has five blades (numbered I–V), and the active site of the MeVINVs is situated inside this module. The motifs NDPNG (Aspartic acid is active site), RDP (Aspartic acid is active site), and WECPD (Glutamic and Cysteine are active sites), which contain active sites were located in the I, III and IV blades, respectively.

**Figure 7:**
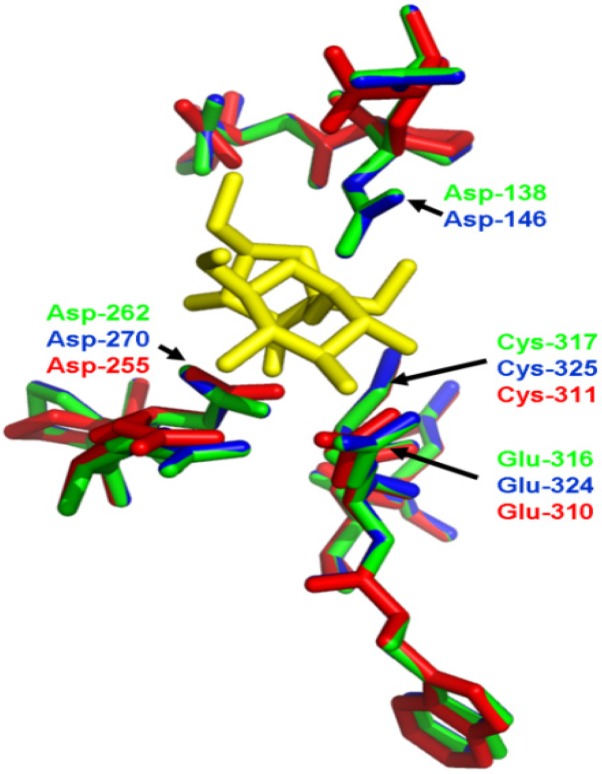
Comparison of the conserved motif in MeVINVs. The residues in MeVINV1, MeVINV2 and MeVINV3 are shown in green, blue and red, respectively. Sucrose molecule is depicted in yellow. Arrows indicate the catalytic residues.

**Figure 8:**
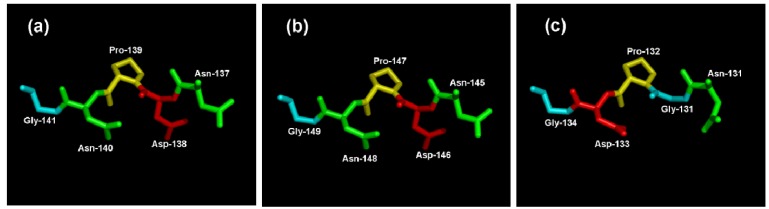
The close-up views of the NDPNG and NGPDG residue structure. (**a**) the structure of NDPNG within MeVINV1; (**b**) the structure of NDPNG within MeVINV2; (**c**) the structure of NGPDG within MeVINV3.

In order to predict the theoretical position of sucrose when binding with MeVINVs, the primary model of MeVINVs was further structurally aligned with *Arabidopsis* AtcwINV1 D239A mutant in complex with sucrose. The motifs NDPNG (NGPDG), RDP and WECVD in MeVINVs were superimposed (Figure 7). The active site residues located in III and IV blades of all three MeVINVs took the same orientation due to the high identity between their predicted active sites (Figure 7). However, the motif of *MeVINV3* in the I blade (NGPDG) was different from the other vacuolar invertases (NDPNG), and the difference in this active site left their structure changed (Figure 8).

### 2.5. The Differential Expression Analysis of MeVINVs in Cassava Organs or Tissues

**Figure 9:**
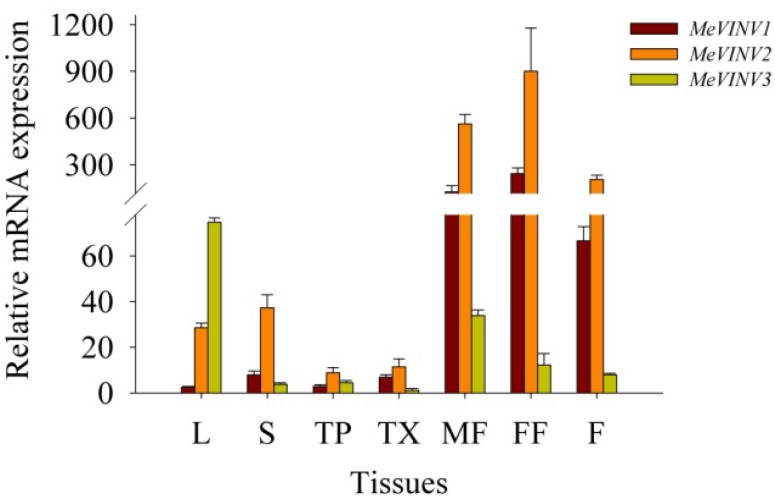
Expression profiles of *MeVINVs* in cassava organs or tissues. The amount of *MeVINV* mRNA was normalized by tubulin mRNA, and the expression of *MeVINV3* in tuber xylem was used as a calibrator. Each value is the mean ± SE of three independent biological replicates. Notes: L, Leaf, S, Stem, TP, Tuber phloem, TX, Tuber xylem, MF, Male flower, FF, Female flower, F, Fruits.

We examined the expression of *MeVINV* sub-family genes in leaves, stems, tuber phloem, tuber xylem, male and female flowers and fruits using qRT-PCR. The results showed that *MeVINVs* were expressed in all the tested tissues, in which the *MeVINV1* and *MeVINV2* were highly expressed in the reproductive organs of male and female flowers and fruits, and weakly expressed in other tissues of leaves, stems, tuber phloem and tuber xylem; while the expression of *MeVINV3* was comparatively higher in leaves and male flowers than in other organs (Figure 9). In the reproductive organs, the highest expression was of *MeVINV2*, followed by *MeVINV1,* and the lowest expression was of *MeVINV3*. In the vegetative organs of leaves and stems, the comparative higher expression in leaves was of *MeVINV3* and *MeVINV2*, and in stems *MeVINV2*; however, all the three genes showed a lower expression in the root tissues of tuber phloem, tuber xylem at 90 days after planting (Figure 9).

### 2.6. The Differential Expression of MeVINVs During Cassava Tuber Root Development

The differential expression of *MeVINV* genes was examined in leaves, tuber xylem and tuber phloem of the cassava plant using qRT-PCR at stages of 90, 135, 180, 225 and 270 days after planting during tuber root development. The cassava plant initially develops tuber roots at 90 days and expands tuber roots with starch accumulation at 135 and 180 days; tuber roots reach maturity at 225, 270 days [31].

**Figure 10:**
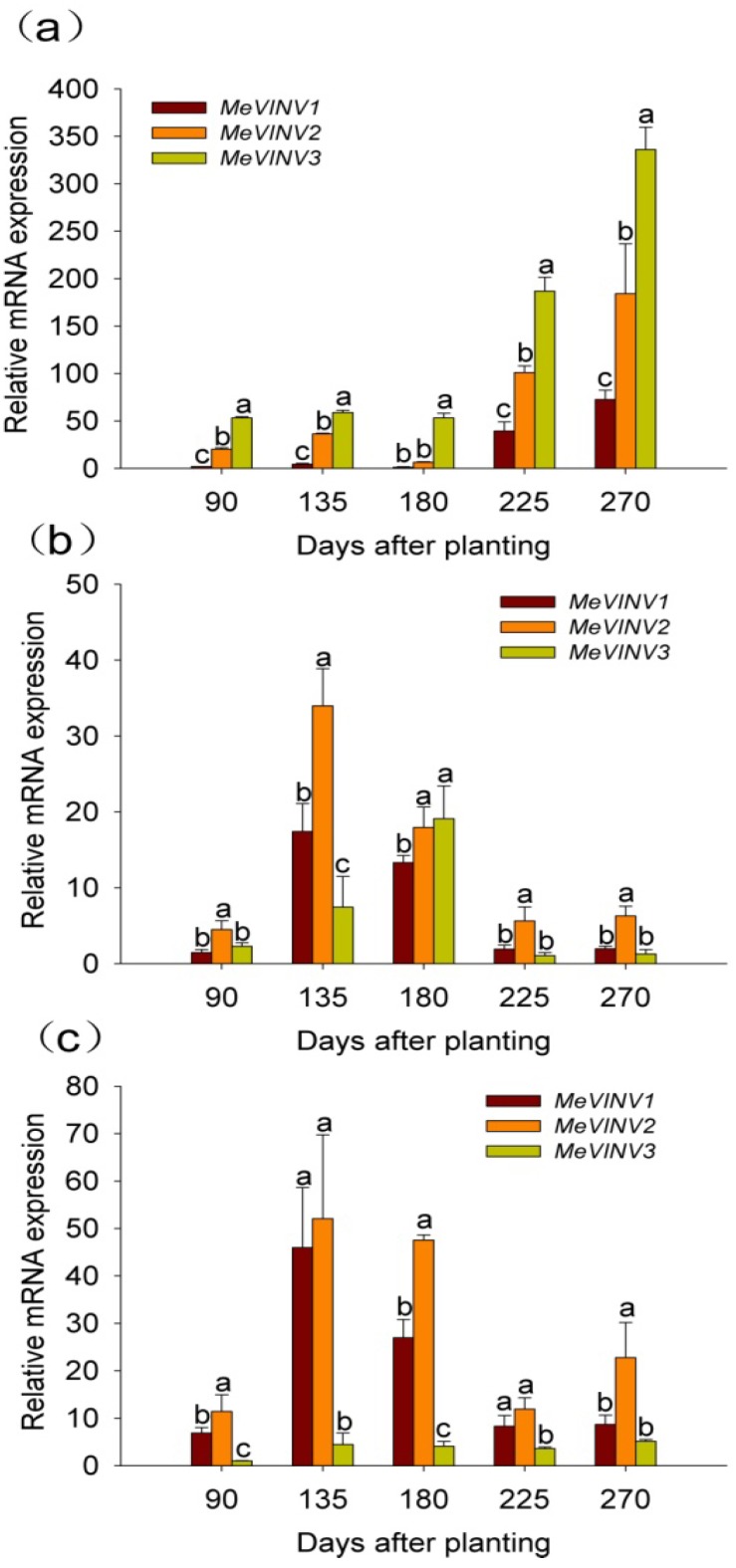
Expression profiles of *MeVINVs* in leaves (**a**), tuber phloem (**b**) and tuber xylem (**c**) at tuber developmental stages in cassava. The amount of *MeVINV* mRNA was normalized by the tubulin mRNA, and the expression of *MeVINV3* in tuber xylem at 90 days was used as a calibrator. Each value is the mean ± SE of three independent biological replicates. Letters on the error bars indicate the significant difference from each gene by ANOVA analysis (*p* < 0.05).

The results showed that the expression of all three *MeVINV* genes in leaves were low at tuber root initial stage (90 days) and expanding stage (135, 180 days), and high at tuber maturity stage (225, 270 days). The highest expression was at the later tuber maturity stage of 270 days. In comparison with the relative RNA transcription levels of the respective *MeVINV* family genes in leaves during cassava tube root development, the results showed that the expression of *MeVINV3* was highest*,* followed by *MeVINV2,* and the lowest expression was of *MeVINV1* (Figure 10a).

The relative mRNA transcription levels of the respective *MeVINV* genes in tuber phloem during the cassava tube root development stages showed that the high expression of all three *MeVINVs* was at tuber expanding stages of 135 and 180 days, and the low expression was at 90 days, 225 days and 270 days (Figure 8b). In comparison to their expression levels in tuber phloem during cassava tuber root development, the results showed that the expression of *MeVINV2* was highest*,* followed by that of *MeVINV1* and *MeVINV3* (Figure 10b)*.*

In tuber xylem, the high expression of *MeVINV1* and *MeVINV2* was at the tuber expanding stages of 135 and 180 days; and the comparative low expression was at 90 days, 225 days and 270 days (Figure 10c). However, the low expression of *MeVINV3* in tuber xylem was found at all stages. In comparison to their expression levels in tuber xylem during cassava tuber root development, the results showed that the expression of *MeVINV2* was higher than *MeVINV1* at all stages (Figure 10c).

### 2.7. Discussion

In the present study, we isolated and characterized three vacuolar invertase genes from the cassava genome. The ORF length and molecular mass of the three *MeVINVs* are similar, ranging from 1,920 bp to 1,983 bp in length, and 71.5 kDa to 73.3 kDa in the predicted molecular mass (Table 1). Their predicted isoelectric pHs are between 4.60 and 5.75, which are consistent with that of the vacuolar invertase proteins in other plants [32]. Signal anchor was found in the N-terminal leader sequence of MeVINVs. The vacuolar invertase is sorted to the vacuole in a form which is anchored to the membrane [28]. The identities of the deduced amino acid sequences of MeVINV family members share 57.49%–84.72% protein identity. Cassava vacuolar invertases can be subgrouped into two subfamilies: The α and β groups. *MeVINV1* and *MeVINV2* are classified as α group, and *MeVINV3* is classified as β group (Figure 5). There are seven exons in all three vacuolar invertases of *MeVINVs*, and these exon-intron structures are consistent with the reported vacuolar invertases in other plants, such as *P. trichocarpa* and *O. sativa* [10,33]. The intron structure of *MeVINV1* and *MeVINV2* is similar and both have their first intron as longest, while in *MeVINV3*, it is the second intron that is longest (Figure 4).

The 3D structural model of MeVINVs shows that the N-terminus consists of a five-bladed β-propeller module, while the C-terminal domain formed a β-sandwich module (Figure 6). The three major conserved motifs, namely NDPNG, RDP and WECVD, are located in the active site of the β-propeller module. This structure is typical of acid invertase protein and implies that MeVINVs has the same structure and catalytic function as other acid invertase proteins. The motifs of NDPNG, RDP and WECVD, which are conserved and essential for catalytic activity in the vacuolar invertase family [34,35], were found in *MeVINV1* and *MeVINV2*, while in *MeVINV3*, in place of the NDPNG motif we found NGPDG, in which the three amino acids GPD are different from those in other vacuolar invertases (DPN). The different amino acid sequences led the 3D structure differently in the active sites of the I blade. It could inactivate the bamboo vacuolar invertase (Bobfruct3) by site-directed mutagenesis the Asp135 (located to NDPNG motif) [35]. In the potato, the pre-mRNA of two vacuolar invertase (CD111 and CD141) genes were susceptible to alternative splicing under cold stress, and lost their mini exon (encode DPN) [36]. Currently, the mini exon GDP replaces DPN in the NDPNG motif in the cassava vacuolar invertase of MeVINV3; the changes on the enzymatic characteristics of MeVINV3 need further study to identify.

The tissue-special expression pattern of *MeVINVs* provides a basis for understanding the function of vacuolar invertase in cassava plant development. Our results showed that *MeVINV1* and *MeVINV2* were highly expressed in reproductive organs, and *MeVINV3* was more expressed in leaves (Figure 9). Other studies have reported that the vacuolar invertases function more in rapidly growing tissues with a high demand for hexoses, such as young ovaries [37], and earlier stages of fruit development [38,39]. The expression pattern implies that *MeVINV1* and *MeVINV2* play a major role in reproductive organs to support hexoses for their growth and development. *MeVINVs* were highly expressed in leaves at the tuber maturity stages (225, 270 days) when starch accumulation in the tuber is reduced, and the transportation of carbohydrate (sucrose) from the leaves to the tuber is decreased (Figure 10a). In plants, it has been reported that vacuolar invertase is involved in futile cycling of sucrose in leaf vacuoles, and plays a buffering role on the pools of sucrose, hexoses and sugar phosphates [26,27]. This implies that at the tuber root maturity stage, most of the carbohydrate (sucrose) is accumulated in leaves, and enters the futile cycling of sucrose in leaf vacuoles where MeVINV1 and MeVINV2 mayplay a main role in this process (MeVINV3 may be a defective invertase). At the early stage of 90 days after planting, all three vacuolar invertases *MeVINVs* were less expressed in tuber roots than in leaves and stems (Figure 9), which indicates that at the early stage, the hexoses from the hydrolyzed sucrose by vacuolar invertases are used for the plant’s rapidly growing vegetative part, such as leaves and stems. During the cassava tuber root development, the tuber phloem is the crucial tissue for sucrose unloading [40,41], and the tuber xylem is the main tissue of cassava starch accumulation [42]. The expression profiles of *MeVINVs* in tuber developmental stages showed that all three *MeVINVs* were highly expressed in tuber phloem at the tuber expanding stage of 135, 180 days, which suggests that all three vacuolar invertases in cassava are involved in phloem unloading of sucrose (Figure 10b). However, in tuber xylem, only *MeVINV1* and *2* were highly expressed at the tuber expanding stage (Figure 10c); thus, *MeVINV1* and *2* may play an important role in starch accumulation.

Bioinformatics and gene expression of *MeVINVs* were studied in this article, suggesting the role of vacuolar invertase in sucrose metabolism of cassava. However, invertase activity can be regulated by post-transcriptional level [43], Such as, in potato tuber, the vacuolar invertase (StvacINV1) was regulated by invertase inhibitor (StInvInh2) during cold-induced sweetening [44]; the vacuolar invertase inhibitor (SolyVIF) of tomato inhibited the vacuolar invertase (TIV-1) and played an important role during tomato plant development [45]. In cassava, we have cloned two speculated vacuolar invertase inhibitors (*MeINH1* and *MeINH2*) [46]. How the cassava vacuolar invertases and inhibitors (*MeVINVs* and *MeINHs*) regulate the sucrose metabolism during tuber root development will further investigated.

## 3. Experimental

### 3.1. Plant Materials

**Figure 11:**
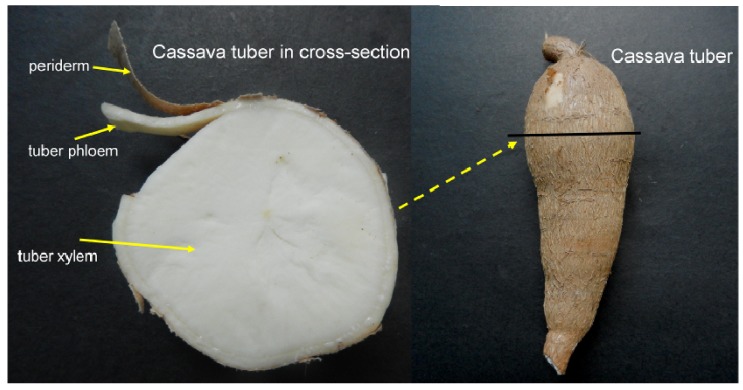
Separation of the cassava tuber phloem and tuber xylem.

Cassava cultivar SC8 (*Manihot esculenta* Crantz no SC8) obtained from the Tropical Crops Genetic Resource Institute (TCGRI, Danzhou, China), Chinese Academy of Tropical Agricultural Sciences (CATAS, Haikou, China), was planted in a field under natural conditions with an average temperature of 23.8 °C. For gene cloning and differential expression analysis in tissues and organs, the plant materials were collected as follows: the leaves, stems, tuber phloem and tuber xylem were collected 90 days after planting; the male and female flowers were collected 200 days after planting; and the fruits were collected 225 days after planting. For differential expression analysis of these genes in source and sink organs during tuber root development, the plant materials were collected as follows: the leaves, tuber phloem and tuber xylem were collected at 90, 135, 180, 225 and 270 days after planting. Tuber phloem and tuber xylem are easily separated by cutting the tuber phloem; we slit the tuber phloem and tuber xylem in the middle part of the tuber (Figure 11). All materials were harvested and stored in liquid nitrogen at −80 °C for RNA isolation.

### 3.2. Molecular Cloning of MeVINVs

A set of gene-specific primers (Table 2) was designed by the primer 5 program based on BLAST analysis of the cassava genome database [47], using the published sequences of vacuolar invertases in *Arabidopsis* and *P. trichocarpa.* Total RNA was extracted using RNAplant Plus reagent (TianGen, Beijing, China). The first-strand cDNA was synthesized from 10 μg of total RNA using the RNA PCR Kit (AMV) Ver.3.0 (TaKaRa, Dalian, China). cDNA was used as template for polymerase chain reaction (PCR) with the primer set designed for each gene. The PCR products were purified from a 1% agar gel and cloned in the pMD18-T vector (TaKaRa). Sequencing was performed using the dideoxynucleotide chain termination method (Sangon Biological Engineering Technology and Services Company, Shanghai, China).

**Table 2:** Gene specific primers used for RT-PCR and real-time RT-PCR.

Gene	Forward primer (5'–3')	Reverse primer (5'–3')
RT-PCR		
*MeCVINV1*	ATATCTAGAATGGCAGACCCCAACC	TATGTCGACAAAGATGAGTTTCACAGT
*MeCVINV2*	ACCCTTCTTCCGTCCTTCTTC	AAATGAGGTAGACTTGGAAAGGTAAG
*MeCVINV3*	TGAGGATCCCCAGCAAATACATGGACTAT	GCCGTCGACCAAAATAGGAGGTGTT
Real-time RT-PCR		
*MeCVINV1*	TTGAGACTAAGAGCCCGAGAAT	AGGACCAGAAGACCGAAGG
*MeCVINV2*	CAGCCTGAGAAGAACTGGATG	GCCAGTCCATACACCATTTTG
*MeCVINV3*	CACCTFTATTTTTCCGTCCTF	ATFCCCAACTTTCACCTTC

### 3.3. Gene Bioinformatics Analysis

Multiple sequence alignments, isoelectric point and molecular weight of the deduced vacuolar invertase protein were analyzed using the DNAman 6.0 program (Lynnon Biosoft, Quebec, QC, Canada). The membrane-spanning domains were predicted using the TMHMM server [48]. A phylogenetic tree was constructed using the neighbor-joining method with a bootstrap value of 1000 in the MEGA 5.2 program. The intron-exon distribution in *MeVINVs* was drawn by the Gene Structure Display Server [49] based on the aligning cDNA sequences of *MeVINVs* with the genomic sequence in the cassava genome database. The three-dimensional (3D) modeling of MeVINVs was predicted by fully-automated protein structure homology modeling [50]. The model returned from the server was further structurally aligned with *Arabidopsis* AtcwINV1 D239A mutant in complex with sucrose (PDB id: 2QQU) using the Pymol software (Delino Scientific, San Carlos, CA, USA) to predict the theoretical position of sucrose when binding with MeVINVs. The catalytic and enzymatically important residues of MeVINVs were also displayed using the Pymol software.

### 3.4. Real-time RT-PCR Analysis

Total RNA was isolated from frozen materials using RNAplant Plus reagent (TianGen). The quantity of RNA was checked by electrophoresis. Reverse transcription was carried out with the PrimeScript™ RT reagent Kit with gDNA Eraser (Perfect Real Time) (TaKaRa) according to the manufacturer’s protocol. The relative mRNA expression of *MeVINVs was analyzed by* quantitative real-time RT-PCR (qRT-PCR) using the qRT-PCR primers given in Table 2. he reactions were performed in a 384-well plate in a volume of 10 μL containing 5 μL 2 × SYBR^®^ Premix Ex Taq II (Tli RNaseH Plus), 0.2 μL ROX Reference Dye (50×), 0.2 μL forward and reverse primers (10 μM), 0.4 μL H_2_O, 4 μL template cDNA (SYBR green reagents were supplied by Takara Dalian, China). The thermal profile for PCR was 95 °C for 60 s, followed by 45 cycles at 95 °C for 5 s, 61 °C for 30 s, final dissociation at 95 °C for 15 s, 60 °C for 15 s and 95 °C for 15 s using the ABI 7900 HT Fast Real-Time PCR System (Applied Biosystems, Foster City, CA, USA). The Ct value (threshold cycle) was defined as the qRT-PCR cycle number that crossed an arbitrarily chosen signal threshold in the log phase of the amplification curve using the thermocycler’s internal software (7900 System SDS software, V2.4, Applied Biosystems). Gene expression was analyzed using the 2^−^^ΔΔCt^ method [51], and the cassava tubulin gene was used as a reference gene [52].

## 4. Conclusions

In summary, three *MeVINVs* were cloned from cassava cultivar SC8, and classified to α (*MeVINV1* and *2*) and β groups (*MeVINV3*). All these vacuolar invertases contain a β-fructosidase and the catalytic site conserved motifs of NDPNG (NGPDG), RDP and WECVD. MeVINVs have similar protein structure to other plant acid invertases. *MeVINV1* and *2* are highly expressed in reproductive organs and tuber roots, considered as sink organs, and may play an important role in starch accumulation. Though *MeVINV3* are highly expressed in leaves, however the mini exon GDP replaces DPN in the NDPNG motif may inactivate this protein; therefore, we speculate that *MeVINV1* and *2* play a role in buffering the pools of sucrose, hexoses and sugar phosphates in leaves, specifically at later stages of plant development.
